# *Radix isatidis* Polysaccharides Inhibit Influenza a Virus and Influenza A Virus-Induced Inflammation via Suppression of Host TLR3 Signaling In Vitro

**DOI:** 10.3390/molecules22010116

**Published:** 2017-01-11

**Authors:** Zhengtu Li, Li Li, Hongxia Zhou, Lijuan Zeng, Tingting Chen, Qiaolian Chen, Beixian Zhou, Yutao Wang, Qiaoyan Chen, Ping Hu, Zifeng Yang

**Affiliations:** 1 State Key Laboratory of Respiratory Disease, National Clinical Research Center for Respiratory Disease, Guangzhou Institute of Respiratory Disease, First Affiliated Hospital of Guangzhou Medical University, (Guangzhou Medical University), Guangzhou 510120, China; tu276025@163.com (Z.L.); zhouhongxia00307@163.com (H.Z.); zwq.1988@163.com (L.Z.); lkting@126.com (T.C.); qiangwei86@163.com (Q.C.); 13710749650@126.com (B.Z.); 2 The First Hospital of Yulin, Yuxi Da Dao Road, Yulin 719000, China; shanxilili79@163.com; 3 Guangdong Provincial Hospital of Traditional Chinese Medicine, Guangzhou University of Traditinal Chinese Medicine, Guangzhou 510180, China; christinecqy@163.com; 4 Shanghai Key Laboratory of Functional Materials Chemistry, School of Chemistry and Molecular Engineering, East China University of Science and Technology, Shanghai 200237, China; 5 Macau University of Science and Technology, AvenidaWai Long, Taipa, Macau 519020, China

**Keywords:** anti-inflammatory, antiviral, polysaccharides, *Radix isatidis*, TLR-3

## Abstract

Influenza remains one of the major epidemic diseases worldwide, and rapid virus replication and collateral lung tissue damage caused by excessive pro-inflammatory host immune cell responses lead to high mortality rates. Thus, novel therapeutic agents that control influenza A virus (IAV) propagation and attenuate excessive pro-inflammatory responses are needed. Polysaccharide extract from *Radix isatidis*, a traditional Chinese herbal medicine, exerted potent anti-IAV activity against human seasonal influenza viruses (H1N1 and H3N2) and avian influenza viruses (H6N2 and H9N2) in vitro. The polysaccharides also significantly reduced the expression of pro-inflammatory cytokines (IL-6) and chemokines (IP-10, MIG, and CCL-5) stimulated by A/PR/8/34 (H1N1) at a range of doses (7.5 mg/mL, 15 mg/mL, and 30 mg/mL); however, they were only effective against progeny virus at a high dose. Similar activity was detected against inflammation induced by avian influenza virus H9N2. The polysaccharides strongly inhibited the protein expression of TLR-3 induced by PR8, suggesting that they impair the upregulation of pro-inflammatory factors induced by IAV by inhibiting activation of the TLR-3 signaling pathway. The polysaccharide extract from *Radix isatidis* root therefore has the potential to be used as an adjunct to antiviral therapy for the treatment of IAV infection.

## 1. Introduction

Influenza is a highly contagious severe respiratory disease that can be caused by influenza A virus (IAV). Seasonal infections with the most common influenza virus strains cause significant mortality; greater than 40,000 deaths and 250,000 hospitalizations annually in the United States alone [1]. Further, the continuous and rapid emergence of new highly pathogenic avian influenza viruses (HPAIV) of the H5N1 subtype, and more recently, pandemic H7N9/H10N8/H5N6 subtypes, pose serious challenges to human and animal health worldwide [2,3,4]. Although vaccination strategies are well-established for influenza prevention and control, lengthy vaccine production cycles limit vaccine availability [5]. Therefore, antiviral drugs are an important primary intervention. However, the rapid development of resistance is a major problem [6,7]. Thus, there is an urgent need to identify novel antiviral drugs to treat influenza.

Infection by IAV is frequently characterized by considerable inflammation that is usually even more pronounced in the case of avian influenza [8,9,10]. It is becoming clear that the morbidity associated with influenza A virus (IAV) infections is a consequence of tissue damage caused by inflammation resulting from the release of pro-inflammatory chemokines and the recruitment of neutrophils, lymphocytes, and particularly mononuclear phagocytes into the alveolar space to limit viral spread [11]. It has been postulated that novel antiviral agents could control IAV propagation, thus attenuating excessive pro-inflammatory responses and limiting symptoms and tissue damage associated with influenza. An increasing number of antiviral drugs influence the host immune response [12], with successful drugs exhibiting both antiviral and anti-inflammatory functions [13]. Based on this concept, traditional Chinese herbal medicine has an innate advantage in that it can target the virus and the host simultaneously, offering more effective inhibition of the host inflammatory immune response induced by IAV. Thus, the development of novel therapeutic agents from traditional Chinese herbal medicine is a promising approach to help combat IAV.

*Radix isatidis*, also known as Ban-Lan-Gen in Chinese, is a traditional Chinese herbal medicine that has traditionally been used for the treatment of influenza, viral pneumonia, mumps, pharyngitis, and hepatitis [14]. Different types of compounds have been isolated from *Radix isatidis* including indirubin, clemastanin B, alkaloids, lignans, and flavonoids [15,16]. Among these, indirubin has been shown to have potent anti-influenza viral activity via inhibition of RANTES (also known as CCL5) expression [17,18]. In our previous study, we found that clemastanin B also exhibited a broad range and selective antiviral activity against various subtypes of IAVs [14]. Furthermore, we reported that lariciresinol-4-*O*-β-d-glucopyranoside exhibited anti-IAV activity and decreased pro-inflammatory cytokine expression via inhibition of the NF-κB pathway in IAV-infected alveolar epithelial (A549) cells [19]. It has also been reported that methanolic extracts of *Radix isatidis* decreased the production of inflammatory mediators in lipopolysaccharide (LPS)-stimulated murine macrophages and in a 12-*O*-tetradecanoyl-phorbol-13-acetate (TPA)-induced ear edema animal model [20]. Of the polysaccharides extracted from *Radix isatidis* root, only have been shown to have antiviral effects by blocking viral attachment [21]. It has not been determined if *Radix isatidis* polysaccharides have antiviral and/or anti-inflammation-induced by IAV. Therefore, here we investigated the potential antiviral and anti-inflammatory activity of the polysaccharides with the aim of developing a novel anti-influenza therapeutic agent.

## 2. Results

### 2.1. Component Analysis of R. isatidis Polysaccharides

In our previous study, we purified the polysaccharide extract from *R. isatidis* root and performed composition analysis [22]. Here, we found that the monosaccharide composition for *R. isatidis* polysaccharides was predominantly mannose, glucose, galactose and arabinose, and the amino acid composition was aspartic acid, glutamic acid serine, histidine, glycine, threonine, arginine, alanine, tyrosine, cystine, valine, phenylalanine, isoleucine, leucine, and lysine. Furthermore, four types of homogeneous polysaccharides were identified among the *R. isatidis* polysaccharides, with M_W_ (Da) of 18,000, 31,000, 36,000, and 82,000 [22].

### 2.2. Anti-Influenza a Virus Activities of R. isatidis Polysaccharides

We investigated the anti-influenza virus activities of *R. isatidis* polysaccharides and found inhibitory effects against different subtypes of influenza virus strains, including human influenza viruses (H1N1 and H3N2) and avian influenza viruses (H6N2 and H9N2), with IC_50_ values ranging from 4.35 ± 0.07 to 28.20 ± 0.49 mg/mL. The inhibitory effect against H3N2 subtype virus was stronger than that against H6N2. These findings suggested that the *R. isatidis* polysaccharides have broad-spectrum antiviral activity against influenza A viruses (Table 1).

Cells were treated with *R. isatidis* polysaccharides, oseltamivir, or 0.5% DMSO (mock) following infection of the indicated influenza virus. Virus titers were determined at 48 hpi by a cytopathic effect (CPE) inhibition assay. Data shown are the mean ± SD for three independent experiments. Note that some of these data were published by our research team, Hu Ping et al. [22].

### 2.3. R. isatidis Polysaccharides Can Effectively Inhibit Human Influenza Virus PR8/H1N1-Induced Cytokine Expression in 16HBE Cells

**Figure 1:**
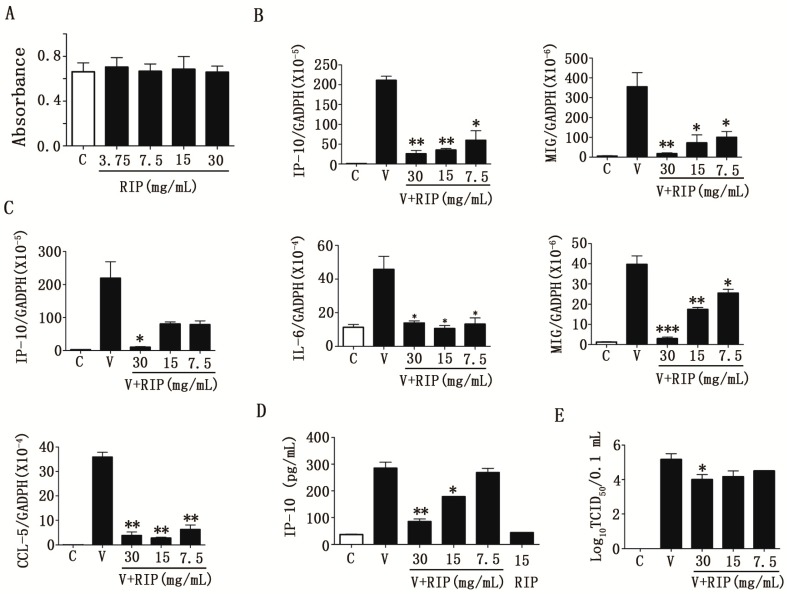
*Radix isatidis* polysaccharides showed anti-inflammation activity in 16HBE cells infected with PR8/H1N1 virus. (**A**) Cell viability was evaluated as described in Materials and Methods and expressed as a percentage of the vehicle control. After mock-infection or infection with PR8 (MOI = 0.1 TCID_50_/cell), 16HBE cells were treated with *R. isatidis* polysaccharides or 0.5% DMSO. Total RNA of the 16HBE cells at 6 h (**B**) or 24 h (**C**) was isolated, and RT-qPCR was performed. Samples were normalised to GAPDH as a control. The protein level of IP-10 was tested by ELISA (**D**), and the virus titer in the supernatant was tested by the CPE method (**E**). RIP: *R. isatidis* polysaccharides. Data are shown as the mean ± SD for three independent experiments. Statistical significance was evaluated using the Student’s *t*-test, relative to the mock-infected cells (* p < 0.05, ** *p* < 0.01, *** *p* < 0.001).

Prior to evaluating the anti-inflammatory activity of *R. isatidis* polysaccharides in cells after influenza virus infection, the cytotoxic effect of the polysaccharides was first tested using an MTT assay. As shown in Figure 1A, no appreciable cytotoxicity in 16HBE cells could be observed at concentrations ranging from 3.75–30 mg/mL when comparing the absorbance levels to those of control cells without drug treatment.

To determine the influence of *R. isatidis* polysaccharides on the expression of pro-inflammatory cytokines/chemokines induced by A/PR/8/34 (H1N1), the mRNA levels of interleukin (IL)-6, IP-10, MIG, and CC chemokine motif ligand 5 (CCL-5) in 16HBE cells were determined by RT-qPCR at 6 and 24 h. As shown in Figure 1B, 30, 15, and 7.5 mg/mL doses of *R. Isatidis* polysaccharides significantly inhibited the mRNA expression levels of IP-10 and MIG (*p* < 0.01) 6 h post-infection. Furthermore, at 24 h post-infection, *R. isatidis* polysaccharides also significantly inhibited the mRNA expression of IL-6 (*p* < 0.05), CCL-5 (*p* < 0.01), and especially MIG (*p* < 0.001), compared with the upregulated expression of all of the tested pro-inflammatory cytokines/chemokines in the PR8-infected cells at a dose of 30–7.5 mg/mL, and it is only has a significant inhibition efficacy in a 30 mg/mL dose for the mRNA expression of IP-10 (*p* < 0.05) (Figure 1C). *R. isatidis* polysaccharides also inhibited the protein expression of IP-10 (*p* < 0.05) at a dose of 30 mg/mL or 15 mg/mL (Figure 1D). At low doses (15 and 7.5 mg/mL), *R. isatidis* polysaccharides suppressed the expression of pro-inflammatory cytokines/chemokines, whereas, at a high dose (30 mg/mL), the polysaccharides inhibited viral replication (Figure 1E). This indicated that *R. isatidis* polysaccharides may be more effective as anti-inflammatory agents than as antiviral agents.

### 2.4. R. isatidis Polysaccharides Can Inhibit Pro-Inflammatory Cytokine Expression Induced by H9N2 Subtype Avian Influenza a Viruses

**Figure 2:**
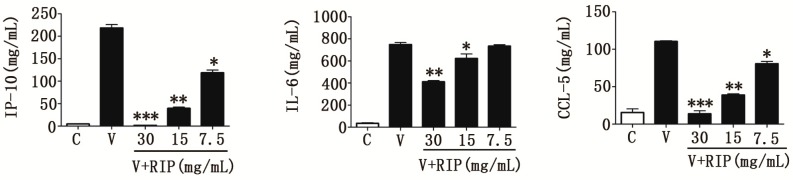
*Radix isatidis* polysaccharides can inhibit cytokine expression induced by H9N2 avian influenza A virus in 16HBE cells. After being mock-infected or infected with H9N2 (MOI = 1), 16HBE cells were treated with *R. isatidis* polysaccharides or 0.5% DMSO. The protein levels of IP-10, IL-6, and CCL-5 were tested by ELISA. RIP: *R. isatidis* polysaccharides. Statistical significance was evaluated using the Student’s *t*-test, relative to the mock-infected cells (* p < 0.05, ** *p* < 0.01, *** *p* < 0.001).

We also investigated the immunomodulatory effect of *R. isatidis* polysaccharides on cytokine induction following avian influenza virus (A/Chicken/Guangdong/1996(H9N2)) infection. As shown in Figure 2, the expression of pro-inflammatory cytokines/chemokines IP-10, IL-6, and CCL-5 induced by H9N2 were markedly reduced. Whilst H9N2-infected cells produced a high level of IP-10 (about 220 pg/mL), infected cells treated with a 30 mg/mL dose of polysaccharides produced 146-fold less (about 1.5 pg/mL) IP-10. Whilst this effect was dose-dependent, even a low polysaccharide dose of 7.5 mg/mL was also effective. Therefore, H9N2-induced cytokine secretion was significantly reduced in the presence of *R. isatidis* polysaccharides in a dose-dependent manner.

### 2.5. R. isatidis Polysaccharides Can Inhibit the Protein Expression Level of TLR3 Induced by PR8/H1N1 Virus in 16HBE Cells

**Figure 3:**
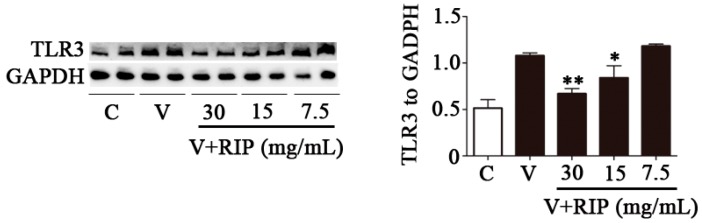
*Radix isatidis* polysaccharides can reduce the expression of TLR3 induced by PR8/H1N1 virus. 16HBE cells were infected with PR8/H1N1 virus (MOI = 1), then treated with *R. isatidis* polysaccharides. After 24 h, Western blotting was performed to assess the TLR3 protein level, and GAPDH protein was used as a control. RIP: *R. isatidis* polysaccharides. Statistical significance was evaluated using the Student’s *t*-test, relative to the mock-infected cells (* p < 0.05, ** *p* < 0.01).

In our previous study, we reported that *R. isatidis* polysaccharides can prevent virus attachment, directly inhibiting virus replication [21]. We also confirmed that *R. isatidis* polysaccharides are more effective anti-inflammatory agents than antiviral agents; however, the anti-inflammatory mechanism remained to be determined. In other research, it was confirmed that polysaccharides can reduce excessive inflammatory responses induced by LPS via the TLR4-NF-ĸB pathway [23]. Thus, we investigated whether *R. isatidis* polysaccharides affect the expression of TLR-3 induced by PR8/H1N1 virus. We found that the expression of TLR-3 induced by PR8 (at 24 h post-infection) was markedly impaired by treatment with *R. isatidis* polysaccharides at higher doses (30 mg/mL or 15 mg/mL) but not at a lower dose (7.5 mg/mL), indicating dose-dependent inhibition (Figure 3).

## 3. Discussion

Our findings provide new evidence that *R. isatidis* polysaccharides can inhibit the replication of influenza viruses, including human and avian influenza viruses. However, these polysaccharides showed even greater efficacy as anti-inflammatory agents in suppressing the inflammation induced by influenza virus through a mechanism that involves the downregulation of host TLR3 at protein level.

The Chinese herbal medicine, *R. isatidis*, has traditionally been used to treat fever and sore throats in the folk of Korea and China [13]. In our previous studies, many active components were found in *R. isatidis* root, including hot water soluble extracts (S-03) [24], clemastanin B [14], and lariciresinol-4-*O*-β-d-glucopyranoside [19], which displayed different antiviral mechanisms and were potential for developing new drugs. In addition, our preliminary study showed that glycopeptides extracted from *R. isatidis* can prevent the attachment of influenza virus to cells [21]. Other studies demonstrated that *R. isatidis* polysaccharides can improve host immunity and induce the production of protective IgG antibodies, which can be used as antiviral vaccine adjuvants [25], or can reduce the expressions of pro-inflammatory factors, such as TNF-α and IL-6, in the LPS-induced murine pneumonia model [20]. In this study, *R. isatidis* polysaccharides were confirmed to be effective in preventing IAV infection (Table 1). Furthermore, *R. isatidis* polysaccharides inhibited the release of pro-inflammatory factors induced by AIV, such as IP-10, IL-6, MIG, and CCL-5 (Figure 1B–D and Figure 2). In fact, *R. isatidis* polysaccharides possessed stronger efficacy in inhibiting inflammatory responses induced by influenza virus (at low doses (15 and 7.5 mg/mL)) than in preventing the virus infection (only at high doses (30 mg/mL)) (Figure 1B–E). Additionally, we found that *R. isatidis* polysaccharides effectively inhibit the release of pro-inflammatory factors induced by LPS (data not shown), which was consistent with other reports [20,26]. Therefore, we suppose that *R. isatidis* polysaccharides are more effective as anti-inflammatory agents than as antiviral agents. With more in-depth research, we have learned that severe influenza is linked to the activation of the host innate immune response, which restricts viral replication and spread. However, prolonged activation can lead to a “cytokine storm” [27]. This virus-induced “cytokine storm” appears to contribute to the severe pathogenesis of IAVs during pandemic outbreaks [28]. Previous in vitro studies have shown that influenza infection induced the production of cytokines IFN-α, tumour necrosis factor (TNF)-α, IL-1, IL-6, and IL-8, and the mononuclear cell attractant chemokines CCL-3/MIP-1α, CCL-4/MIP-1β, CCL-2/MCP-1, CCL-7/MCP-3, CXCL-10/IP-10, and CCL-5/RANTES in human monocytes, epithelial cells, and rat alveolar or murine macrophages [29]. Human research has found that IL-6 and IP-10 are inflammatory markers of severe avian influenza virus H7N9 infection [30]. In the case of human infection with avian influenza virus H5N6, we found that MIG and IP-10 played a critical role in the development of severe respiratory disease [3], which was consistent with the findings in H5N1, H7N9, and severe H1N1 pdm09 patients [9,10,31]. Thus, IP-10, IL-6, MIG, and CCL-5 may be important cytokines in influenza virus infection and may indicate both the severity of disease and drug treatment efficacy during influenza virus infection. Furthermore, in our study, we found that *R. isatidis* polysaccharides can effectively inhibit the expression of IP-10, IL-6, MIG, and CCL-5 induced by human influenza virus (PR8/H1N1) (Figure 1B–D) and avian influenza virus (H9N2) (Figure 2), indicating that these polysaccharides are a promising candidate anti-influenza virus drug with effective anti-inflammation activity.

In our previous study (in the Journal of Ethnopharmacology), we found that, in fruit polysaccharides from *Schisandra chinensis,* different polysaccharides displayed different monosaccharide compositions. Furthermore, we found different anti-inflammatory and antiviral efficacy between *R. isatidis* polysaccharides and *S. chinensis* fruit polysaccharides (data not shown), indicating that different monosaccharide compositions confer different functional properties. Further research is needed, but monosaccharide composition should be carefully considered in the design of polysaccharide drugs to treat influenza virus infection.

Toll-like receptor (TLR)-3 is a member of a conserved family of innate immune recognition receptors that have key roles in detecting microbes, initiating innate immune responses and linking innate and adaptive immunity. TLR-3 is also a major transducer of cell signaling generated by dsRNA [32]. TLR3 has been implicated in the detection of many RNA viruses and in altering the pathogenesis of airway disease resulting from respiratory virus such as IAV and respiratory syncytial virus [32,33]. In our study, we found that PR8/H1N1 virus upregulated the expression of TLR3 (Figure 3). Furthermore, following treatment with *R. isatidis* polysaccharides, TLR3 induced by PR8/H1N1 virus was reduced (Figure 3). From this, we speculate that the underlying mechanism of action of the polysaccharides is to impair the upregulated expression of pro-inflammatory cytokines/chemokines induced by influenza virus by inhibiting the expression of host TLR-3. However, in addition to TLR3, there is another influenza virus pathogen recognition receptor, namely RIG-I-like receptor [34]. Further studies are required to determine whether *R. isatidis* polysaccharides also affect the expression of RIG-I-like receptor to reduce the inflammatory reaction induced by influenza virus.

Whilst our data is promising, there were some limitations in our study. The effective dose of *R. isatidis* polysaccharides was relatively high, but it was comparable to other Chinese herbal medicine, such as Lianhua-Qingwen [35]. Furthermore, studies in animal models are needed to further characterize the effectiveness of the *R. isatidis* polysaccharides derivatives on IAV-infection treatment.

## 4. Materials and Methods

### 4.1. Plant Material and Preparation of the Polysaccharide

The roots of *Radix isatidis* (*R. isatidis*), cultivated in Good Agricultural Practice (GAP) farms in Fuyang (An-Hui Province, China) were obtained from Hutchison Whampoa Guangzhou Baiyunshan Chinese Medicine Co., Ltd., Guangzhou, China. The herbarium specimen was authenticated by Professor Ye Hua Gu at the Chinese Medicine Research Institute. The isolation and purification of *Radix isatidis* polysaccharides and determination of their compositions was performed as previously described [22].

### 4.2. Cells and Viruses

The human bronchial epithelial cell line (16 HBE) and Madin–Darby Canine Kidney (MDCK) cells were purchased from the Cell Bank of the Chinese Academy of Sciences (Shanghai, China; Art. No.: 16HBE: CBP60550; MDCK: CBP60561). Seasonal human influenza viruses including influenza A virus H1N1 (A/PR/8/34), H1N1pd2009 (A/Guangzhou/GIRD07/09), influenza A virus H3N2 (A/Aichi/2/68) were purchased from the CDC and avian influenza A virus H6N2 (A/Duck/Guangdong/1994), and H9N2 (A/Chicken/Guangdong/1996) were gifted from South China Agricultural University (Guangdong province of China). These viruses were propagated in the allantoic cavity of 9-day-old embryonated chicken eggs for 48 h at 35 °C, then 12 h at 4 °C, after which the viruses were harvested and preserved at −80 °C prior to use.

### 4.3. Cytotoxicity Assay

The cytotoxic effect of the polysaccharide on 16HBE cells and MDCK cells was assessed using an MTT assay. Briefly, the 16HBE and MDCK cells (100 μL per well at a density of 1 × 10^5^ cells/mL) were seeded onto 96-well plates for overnight incubation. Then, cells were cultured in the presence or absence of polysaccharide at varying concentrations for 48 h. After 48 h, the culture medium was removed and washed with phosphate-buffered saline (PBS). The filtered MTT solution (0.5 mg/mL) was added to each well, the plate was incubated for 4 h at 37 °C after incubation, the supernatants were aspirated, and the formazan crystals were dissolved in 200 μL of DMSO. The absorbencies were measured at a wavelength of 570 nm using a 96-well microplate spectrophotometer (Molecular Devices, Sunnyvale, CA, USA).

### 4.4. Cytopathic Effect (CPE) Inhibition Assay

MDCK cells (100 µL, 2 × 10^4^ cells/well) were seeded into each well of a 96-well plate and cultured at 37 °C under 5% CO_2_ for 20–24 h. For the anti-influenza activity assay, MDCK cells were inoculated with a total of 100 TCID_50_ of the influenza virus infection titer and incubated at 34 °C for 2 h. The cells were then washed and cultured for 48 h at 37 °C under 5% CO_2_ in the presence of 100 μL of the polysaccharide or oseltamivir (Sigma-Aldrich, St. Louis, MO, USA) in MEM supplemented with 2 μL/mL TPCK-trypsin (Sigma). After incubation, the CPE of the virus-infected cells was observed microscopically. The IC_50_ of the virus-induced CPE was detected by the method of Reed–Muench [36]. Each value was an average from three independent experiments. The selectivity index (SI) was calculated by the TC_50_/IC_50_ ratio. For the progeny virus, the inhibition effects of the polysaccharide were tested by the CPE method. After 24 h treatment with the polysaccharide, the cell supernatant was collected, and 100 µL were added along with 2 μL/mL TPCK-trypsin into the prepared MDCK cells. After 48 h, the virus-induced CPE was detected by the method of Reed–Muench.

### 4.5. Cell Treatment and Inflammatory Cytokine Analysis

16HBE cells were plated at a density of 1 × 10^6^ cells/well in six-well plates. After overnight incubation, the cells were washed with PBS and infected with influenza virus A/PR/8/34(H1N1) or A/Chicken/Guangdong/1996 (H9N2) for 2 h at 37 °C. Then, the inoculum was removed and the cells were incubated with serum-free DMEM medium containing various concentrations of *R. isatidis* polysaccharides for 6 or 24 h. Total RNA was extracted from 16HBE cells using TRIzol reagent (Invitrogen, Carlsbad, CA, USA) and 1 μg of total RNA was reverse transcribed into cDNA using the Prime-Script RT kit (Takara, Shiga, Japan) following the manufacturer’s protocol. Quantification of the analyses by quantitative PCR (qPCR) was performed using an ABI7500 Real-Time PCR System. The RT-qPCR primers and probes for analyses are listed in Table 2. Quantitative amplification conditions were as follows: denaturation at 95 °C for 30 s, followed by 40 cycles of denaturation at 95 °C for 5 s, and annealing and elongation at 60 °C for 40 s. The protein levels of inflammatory cytokines in the cell supernatant were tested by an ELISA kit purchased from eBioscience (San Diego, CA, USA).

### 4.6. Western Blot Analysis

Cells were homogenized in RIPA lysis buffer containing a 1% protease inhibitor cocktail (Sigma-Aldrich), 5 μM EDTA, and 200 μM 4-(2-aminoethyl) benzenesulfonyl fluoride hydrochloride to extract the total protein. Equal amounts of protein homogenates in the samples were separated by SDS-PAGE (Bio-Rad, Hercules, CA, USA), transferred onto polyvinylidene difluoride membranes (pore size 0.45 μM, Bio-Rad), blotted with human monoclonal antibody against TLR3 (1:1000, Cell Signaling Technology, Beverly, MA, USA) or GAPDH (1:1000, Cell Signaling Technology), and probed with the corresponding peroxidase-conjugated secondary antibodies (KPL, Gaithersburg, MD, USA). Signals from bound antibodies were detected using an Immun-Star HRP Chemiluminescent kit (Bio-Rad).

### 4.7. Statistical Analysis

Statistical analyses were carried out using SPSS Base 18.0 (IBM, Armonk, NY, USA) for Windows (SPSS). Comparisons among treatment groups were determined by one-way ANOVA followed by LSD-t post-hoc test for multiple comparisons. Data are presented as mean values with the standard deviations indicated (mean ± SD). Differences were considered to be statistically significant when the *p*-value was less than 0.05.

## 5. Conclusions

**Figure 4:**
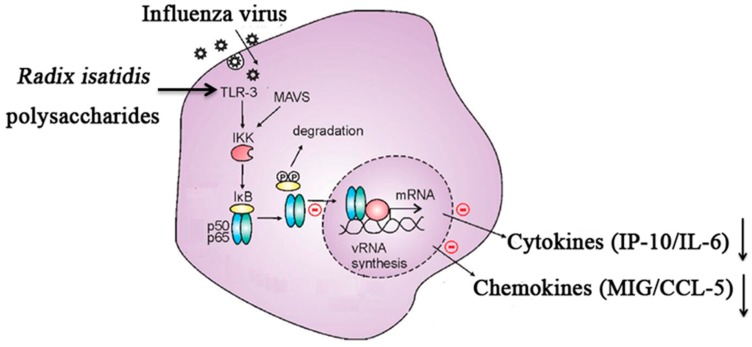
Schematic diagram of the mechanism of suppression of influenza virus-induced inflammation by *Radix isatidis* polysaccharides.

In summary, we found that *R. isatidis* polysaccharides can effectively inhibit the replication of influenza virus, including human and avian influenza viruses. Furthermore, these polysaccharides display even greater efficacy against the inflammatory reaction induced by influenza virus, possibly via inhibition of the host TLR3 signaling pathway (Figure 4). These results provide a scientific basis for considering *R. isatidis* polysaccharides as a new drug candidate for the treatment of influenza virus infection.

## Figures and Tables

**Table 1:** Anti-influenza virus activities of the polysaccharides in vitro.

Virus Type and Strain	Polysaccharide		Oseltamivir			
	TC_50_ (mg/mL)	IC_50_ (mg/mL)	SI	TC_5_ (mg/mL)	IC_50_ (mg/mL)	SI
A/PR/8/34 (H1N1)	>40	20.48 ± 0.31	>1.95	>0.312	0.000238 ± 0.000015	>1000
A/Guangzhou/GIRD07/09 (H1N1)	>40	8.47 ± 0.07	>4.72	>0.312	0.000194 ± 0.000009	>1000
A/Aichi/2/68 (H3N2)	>40	4.35 ± 0.05	>9.20	>0.312	0.00138 ± 0.00017	>100
A/Duck/Guangdong (H6N2)	>40	28.20 ± 0.49	>1.42	>0.312	0.00537 ± 0.00019	>50
A/Chicken/Guangdong/1996 (H9N2)	>40	20.57 ± 0.25	>1.94	>0.312	0.00466 ± 0.00010	>50

**Table 2:** Primers and probes used in this study.

Gene	Primers and Probe	Sequence (5′→3′)
IL-6	Forward	5′-CGGGAACGAAAGAGAAGCTCTA-3′
	Reverse	5′-CGCTTGTGGAGAAGGAGTTCA-3′
	Probe	5′-FAM-TCCCCTCCAGGAGCCCAGCT-3′TAMRA
IP-10	Forward	5′-GAAATTATTCCTGCAAGCCAATTT-3′
	Reverse	5′-TCACCCTTCTTTTTCAT-TGTAGCA-3′
	Probe	5′-FAM-TCCACGTGTTGAGATCA-3′MGB
MIG	Forward	5′-TCTTGCTGGTTCTGATTGGAGTG-3′
	Reverse	5′-GATAGTCCCTTGGTTGGTGCTG-3′
	Probe	5′-FAM-CAGGAACAGCGACCCTTTCTCACTACTGG-3′BHQ-1
CCL-5	Forward	5′-CAGCAGTCGTCTTTGTCACC-3′
	Reverse	5′-GTTGATGTACTCCCGAACCC-3′
	Probe	5′-FAM-CGCCAAGTGTGTGCCAACCC-3′TAMRA
GAPDH	Forward	5′-GAAGGTGAAGGTCGGAGTC-3′
	Reverse	5′-GAAGATGGTGATGGGATTTC-3′
	Probe	5′-FAM-CAAGCTTCCCGTTCTCAGCC-3′TAMRA

## Abbreviations

IAV	Influenza A virus
RIP	*Radix isatidis* polysaccharides
TLR-3	Toll-like receptor-3
CPE	Cytopathic effect
SI	Selectivity index
